# A Pathomics-Based Prognostic Model for Disease-Free Survival in Resected Gastric Cancer

**DOI:** 10.3390/cancers18060993

**Published:** 2026-03-19

**Authors:** Liyun Zheng, Zhiying Jin, Fazong Wu, Shiman Zhu, Yeyu Zhang, Li Chen, Wanbin Chen, Chaoming Huang, Lingyi Zhu, Shiji Fang, Zijian Zhu, Qi Huang, Minjiang Chen, Zhongwei Zhao, Weiwen Li, Shimiao Cheng

**Affiliations:** 1 Zhejiang Key Laboratory of Imaging and Interventional Medicine, The Fifth Affiliated Hospital of Wenzhou Medical University, Lishui 323000, China; zhengliyun@wmu.edu.cn (L.Z.); 2025112028167@stu.hznu.edu.cn (F.W.); 2025112028168@stu.hznu.edu.cn (S.Z.); lxy2001@wmu.edu.cn (L.C.); 202411122911023@zcmu.edu.cn (W.C.); 24021051417@usx.edu.cn (C.H.); zhulingyi@alu.zcmu.edu.cn (L.Z.); fangshiji@wmu.edu.cn (S.F.); 2371062525@wmu.edu.cn (Z.Z.); 202311122911728@alu.zcmu.edu.cn (Q.H.); minjiangchen@wmu.edu.cn (M.C.); zhongwei-zhao@wmu.edu.cn (Z.Z.); 2 Cancer Center, The Fifth Affiliated Hospital of Wenzhou Medical University, Lishui 323000, China; jzy241102329@wmu.edu.cn (Z.J.); 2025112028170@stu.hznu.edu.cn (Y.Z.); 3 Department of Radiology, The Fifth Affiliated Hospital of Wenzhou Medical University, Lishui 323000, China; 4 Clinical College of The Affiliated Central Hospital, School of Medicine, Lishui University, Lishui 323000, China

**Keywords:** gastric cancer, pathomics, prognostic model, disease-free survival, nomogram, clinical variables

## Abstract

Objectives: This study aims to develop and validate a prognostic risk model by integrating pathomics features with clinical variables to predict disease-free survival (DFS) in patients with gastric cancer (GC). Methods: Patients with GC who were pathologically diagnosed and subsequently treated with curative gastrectomy and D2 lymphadenectomy at the Fifth Affiliated Hospital of Wenzhou Medical University between January 2017 and April 2023 were retrospectively enrolled and assigned to a training cohort (*n* = 275) and an independent validation cohort (*n* = 118). Pathomics features were extracted from pathological images, and LASSO-Cox regression was used to identify pathomics features significantly associated with DFS. The selected pathomics features were integrated with clinical factors to create a prognostic model. Predictive accuracy was evaluated using time-dependent ROC analysis, and the model’s performance was compared with the clinic-only and pathomics-only models. A nomogram was constructed to provide individualized DFS predictions. Results: A total of 16 pathomics features were selected, and the cut-off for the pathomics scores was set at 0.27. High-risk patients exhibited significantly worse DFS compared to low-risk patients in both the training cohort (HR = 4.57, 95% CI: 3.118–6.697, *p* < 0.0001) and the validation cohort (HR = 2.264, 95% CI: 1.255–4.083, *p* < 0.0001). The clinic–pathomics model demonstrated strong predictive performance in both cohorts, with AUCs for 1-, 3-, and 5-year survival of 0.832, 0.821, and 0.851 in the training cohort, and 0.671, 0.702, and 0.682 in the validation cohort. The nomogram, incorporating the pathomics score, T stage, differentiation degree, and ECOG performance status, showed high calibration accuracy, as confirmed by calibration plots, and outperformed both the clinic-only and pathomics-only models in decision curve analysis. Conclusions: A clinic–pathomics model integrating pathomics features with clinical data provides a reliable tool for DFS prediction in patients with GC, which facilitates individualized DFS predictions and personalized treatment strategies.

## Simple Summary

Gastric cancer has a high postoperative recurrence rate, and traditional staging systems cannot accurately predict individual recurrence risk. Pathomics can extract quantitative features from pathological slides to reflect tumor biological characteristics, but there is a lack of reliable prognostic models combining pathomics and clinical data. This study aimed to develop and validate a disease-free survival prediction model for postoperative gastric cancer patients by integrating pathomics features and clinical factors. The final model showed better predictive performance, and a practical nomogram was built. This study provides a valuable tool for individualized risk stratification and postoperative management, and promotes the clinical translation of pathomics in gastric cancer.

## 1. Introduction

Gastric cancer (GC) ranks as the fifth most common cancer globally and the third leading cause of cancer-related deaths. Approximately a million new cases are diagnosed annually, and 650,000 people have died from the disease worldwide [1]. Despite advancements in diagnostic and treatment techniques, the prognosis for patients with GC remains poor. Surgical treatment is one of the main methods for curing GC, particularly for early-stage patients. Gastrectomy significantly improves survival rates. However, the postoperative recurrence rates remain high, leading to an unfavorable prognosis. The rates are estimated to range from 14% to 60%, even with the application of adjuvant chemotherapy, immunotherapy, targeted therapy, and radiotherapy to complement gastrectomy [2]. Several clinical and pathological factors have been identified as contributors to postoperative recurrence, including tumor size and depth, lymph node metastasis, histological grade, perineural invasion, vascular invasion, surgical margin status, tumor location, and patient performance status [3,4]. Nevertheless, accurately predicting individual recurrence risk remains challenging due to the complexity of tumor biology. Conventional prognostic models often fail to fully capture this complexity, limiting their ability to provide precise risk assessments.

The TNM staging system is the most widely used framework for assessing tumor burden and guiding treatment strategies in patients with GC [5,6]. It is particularly valuable for evaluating local tumor progression and predicting early recurrence. However, the TNM system has inherent limitations, as it primarily focuses on macroscopic anatomical features without incorporating tumor microenvironment characteristics, biological behavior, or heterogeneity. Thus, patients at the same TNM stage may experience markedly different clinical outcomes, highlighting the need for more individualized and biologically informed prognostic tools. To overcome these limitations, several prognostic models incorporating clinical and pathological variables have been proposed to improve risk stratification and recurrence prediction [7]. However, most of these models still rely on features derived from traditional pathology and clinical records, which may not fully reflect the underlying tumor biology. Increasing evidence indicates that tumor heterogeneity, the immune microenvironment, and molecular alterations play critical roles in disease progression and treatment response, yet these factors are often under-represented in standard models.

With the rapid development of high-throughput omics technologies, clinical–omics-based prognostic models have shown great promise in improving predictive performance. These models integrate multi-dimensional data such as genomic, transcriptomic, proteomic, and metabolomic profiles with clinical parameters to provide a more comprehensive view of tumor biology. Studies have demonstrated that such integrative models can outperform traditional systems in predicting recurrence and survival in patients with GC [8,9]. However, the high cost, technical complexity, and limited accessibility of omics platforms hinder their routine clinical implementation. Artificial-intelligence-driven radiomics has also emerged as a non-invasive method for extracting quantitative features from medical imaging to predict clinical outcomes [10,11,12]. Additionally, radiomics signatures derived from CT or MRI scans have been explored for early recurrence prediction and treatment response assessment in GC [12,13]. Radiomics faces also challenges related to imaging standardization, reproducibility, and interpretability of features, which currently limit its generalizability and clinical utility.

More recently, pathomics, the high-throughput extraction and analysis of quantitative features from digital histopathological slides, has gained attention as a cost-effective and interpretable tool for tumor characterization [14,15]. Pathomics leverages routine hematoxylin and eosin (H&E)-stained whole-slide images (WSIs), which are widely available in clinical practice [14,16]. By quantifying morphological features such as nuclear size, shape, texture, chromatin pattern, spatial distribution, and cell density, pathomics can capture subtle tumor heterogeneity and structural alterations that are often overlooked by human observers. This approach minimizes subjectivity and facilitates objective, reproducible feature extraction. Several recent studies have demonstrated the value of pathomics in the prognostication of GC [14,17]. Chen et al. [18] developed a pathomics-based nomogram to predict peritoneal recurrence in patients with GC with serosal invasion, achieving high-accuracy predictive performance, with a C-index of 0.892. Han et al. [19] developed a pathomics model based on H&E slides that predicts the infiltration degree of activated CD4 memory T cells and provides novel prognostic markers for precise stratification and individualized prognosis in GC. Lou et al. [11] proposed a pathomics-based signature as an independent prognostic factor that could guide adjuvant treatment decisions and identify patients who might benefit from chemotherapy or immunotherapy. These findings support the clinical potential of pathomics for risk stratification and treatment optimization in patients with GC.

In this paper, we propose a pathomics-based model for predicting disease-free survival (DFS) in patients with GC who have undergone curative gastrectomy. Pathomics features were extracted from hematoxylin and eosin (H&E)-stained whole-slide images, and those significantly associated with DFS were identified. In addition, relevant clinicopathologic variables were integrated with selected pathomics features to construct a combined clinical–pathomics model. The predictive performance of the model was evaluated in both training and validation cohorts. We hypothesized that the integration of pathomics features with traditional clinical parameters would provide a more accurate and individualized prediction of postoperative recurrence. This integrative approach may offer valuable insights for guiding personalized follow-up strategies, improving recurrence surveillance, and optimizing adjuvant treatment decisions in patients with GC.

## 2. Patients and Methods

### 2.1. Study Design

**Figure 1:**
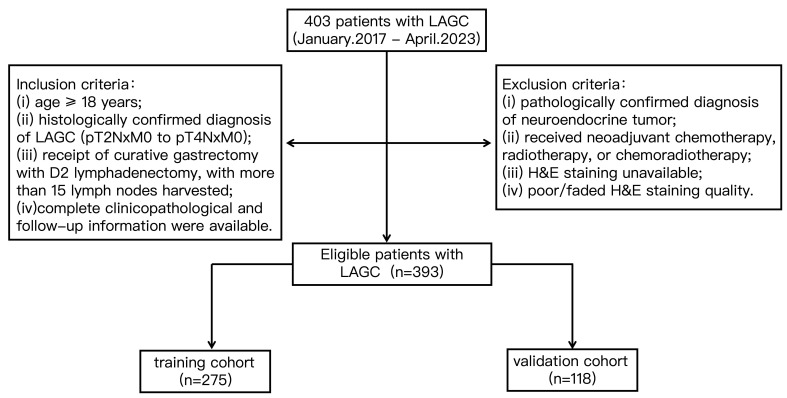
Flowchart of patient screening.

This study was single-center retrospective study and approved by the Institutional Review Board and Human Ethics Committee of the Fifth Affiliated Hospital of Wenzhou Medical University. Patients with GC were pathologically diagnosed and subsequently treated with curative gastrectomy with D2 lymphadenectomy at the Fifth Affiliated Hospital of Wenzhou Medical University between January 2017 and April 2023. The clinical data gathered included age, sex, Eastern Cooperative Oncology Group performance status (ECOG), carcinoembryonic antigen (CEA) level, carbohydrate antigen 19-9 (CA19-9) level, tumor location, tumor size, pT stage, pN stage, pTNM stage, and histological differentiation. In addition, hematoxylin and eosin (H&E)-stained WSIs of GC tissue were obtained. Finally, a total of 393 patients with locally advanced GC who underwent curative gastrectomy were retrospectively included and randomly divided into a training cohort (*n* = 275) and an internal validation cohort (*n* = 118) (Figure 1). DFS was strictly defined as the time interval from the date of curative gastrectomy with D2 lymphadenectomy to the first documentation of any disease recurrence, metastasis, or cancer-related death, whichever occurred first. All patients were followed up starting from the date of curative gastrectomy with D2 lymphadenectomy until the occurrence of study endpoints or the closure of the study period.

### 2.2. Inclusion and Exclusion Criteria

Patients were enrolled in this study based on the following inclusion criteria: (1) age ≥ 18 years; (2) histologically confirmed diagnosis of locally advanced GC (LAGC), defined as pathological stages pT2NxM0 to pT4NxM0 according to the 8th Edition of the American Joint Committee on Cancer (AJCC) staging system; (3) receipt of curative gastrectomy with D2 lymphadenectomy, with more than 15 lymph nodes dissected; and (4) availability of complete clinicopathological information and follow-up data. Patients were excluded if they met any of the following conditions: (1) pathologically confirmed diagnosis of neuroendocrine tumor; (2) receipt of neoadjuvant chemotherapy, radiotherapy, or chemoradiotherapy; (3) absence of hematoxylin and eosin (H&E)-stained whole-slide images; or (4) poor quality of H&E-stained slides that did not meet the requirements for pathomics analysis.

### 2.3. Hematoxylin and Eosin (H&E) Imaging

Tumor tissues preserved as formalin-fixed, paraffin-embedded (FFPE) samples were collected. Then, the samples were sectioned in slices 4–5 μm thick and stained with H&E according to standard histopathological protocols. The stained slides were then digitized using a whole-slide scanner (KF-PRO-005, Kibfo ScanScope Scanner, Ningbo Jiangfeng Bio-Information Technology, Ningbo, China) at 40× magnification to generate high-resolution whole-slide images (WSIs). All WSIs were saved in JPEG or TIFF format for subsequent image analysis. Only slides with sufficient staining quality and clear visualization of tumor regions were included. Two senior pathologists with more than 10 years of specialized diagnostic experience in gastric cancer independently reviewed each WSI to identify and manually outline representative tumor parenchyma regions, with strict exclusion of normal gastric mucosa, extensive necrotic areas, non-tumor stromal tissue, and marginal inflammatory infiltration zones to ensure the annotated regions exclusively contained tumor cells with typical malignant morphological features. Inter-observer agreement for annotation was assessed using Cohen’s kappa coefficient, showing a high level of consistency (κ = 0.85, *p* < 0.001); any annotative discrepancies were resolved by joint consensus of the two pathologists. To minimize selection bias, five representative, non-overlapping tiles (512 × 512 pixels each) containing the most tumor-rich areas were randomly extracted from the manually annotated tumor parenchyma regions of each whole-slide image.

### 2.4. Pathomics Feature Extraction

CellProfiler (version 4.1.3), open-source image analysis software, was used to extract the quantitative pathomics features from the selected images. Briefly, H&E-stained images were first decomposed into hematoxylin and eosin grayscale components using the “UnmixColors” module. In parallel, full-color H&E images were also converted to grayscale via the “ColorToGray” module, employing the “Combine” strategy to enable subsequent quantitative analysis. To assess image quality and intensity characteristics, the “MeasureImageQuality” and “MeasureImageIntensity” modules were applied to both grayscale H&E and the individual hematoxylin and eosin channels. The pixel-level colocalization and correlation between the two stains were evaluated using the “MeasureColocalization” module, enabling the quantification of spatial overlap and signal coherence across the entire image. In addition, the “MeasureGranularity” module was used to extract texture-related features by analyzing spatial patterns at varying scales, generating a granularity spectrum of up to 16 levels to reflect the structural heterogeneity within the tissue.

### 2.5. Pathomics Feature Selection

All extracted features were standardized using z-score normalization to minimize the impact of the differences in measurement scales. A structured two-step approach was applied to identify the most relevant pathomics features associated with DFS. The maximum relevance minimum redundancy (mRMR) algorithm was employed to remove redundant features by discarding those with high inter-feature correlations (Pearson’s r > 0.8). A least absolute shrinkage and selection operator (LASSO)-Cox regression analysis was performed to further reduce dimensionality and identify the most predictive features. The optimal regularization parameter (lambda) was determined through five-fold cross-validation, and the value corresponding to the minimum cross-validated error was selected. This combined mRMR and LASSO-Cox approach capitalizes on their complementary strengths: mRMR first eliminates highly correlated redundant features to reduce dimensionality and avoid collinearity interference, while LASSO-Cox regression further screens features by linking them directly to the DFS outcome, penalizing non-prognostic feature coefficients to zero to identify the most predictive ones. This two-step strategy ensures the selected features are both non-redundant and clinically relevant for prognosis, enhancing the robustness of the subsequent model.

### 2.6. Pathomics Prediction Model Establishment and Performance

The selected pathomics feature were integrated into a Cox proportional hazards model to establish a pathomics prediction model. Then, the pathomics score was calculated for each patient. A cut-off pathomics score were subsequently determined in the training cohort using X-tile software (version 3.6.1, Yale University School of Medicine), and all the patients were assigned to high- and low-risk groups. The discriminative capacity of the pathomics score in both the training and validation datasets was evaluated using confusion matrix visualization to assess classification performance.

### 2.7. Clinical–Pathomics Prediction Model Establishment and Performance

To enhance the predictive accuracy of DFS, clinical variables were incorporated with the pathomics model to establish a clinical–pathomics prediction model. Initially, univariable Cox regression analysis was performed to identify the clinical factors associated with DFS. A multivariable Cox regression model was developed based on the variables with a *p*-value less than 0.05 in the univariable analysis to determine the final set of independent clinical predictors of DFS. The clinical–pathomics prediction model was subsequently developed. The predictive performance of the clinical–pathomics prediction model was assessed using time-independent receiver operating characteristic (ROC) curves in both the training and validation cohorts. Calibration curves and decision curve analysis (DCA) were also used to evaluate the model’s calibration and clinical utility. Finally, a nomogram was constructed to visually represent the prediction model, enabling individualized risk stratification and estimation of DFS probabilities.

### 2.8. Statistical Analysis

R software (version 3.6.3), Python (version 3.7.0), and SPSS (version 26.0) were used. Either Student’s *t* test or the Mann–Whitney *U* test was used to compare differences in continuous variables. The chi-square test or Fisher’s exact test was used to compare differences in categorical variables. Kaplan–Meier survival analysis with the log-rank test was employed to evaluate differences in DFS. Hazard ratios (HRs) with 95% confidence intervals (CIs) were estimated using Cox proportional hazards regression analysis. Statistical significance was defined as a *p*-value less than 0.05.

## 3. Results

### 3.1. Baseline Characteristics of Study Population

The baseline clinicopathological characteristics of the patients in the training cohort (*n* = 275) and validation cohort (*n* = 118) are summarized in Table 1. No statistically significant differences were observed between the cohorts for any clinical variable (*p* > 0.05), indicating comparability. The mean age was 63.89 ± 10.21 years in the training cohort and 64.19 ± 10.04 years in the validation cohort (*p* = 0.794).

The median tumor size was 4.0 cm (IQR: 2.5–6.0) in the training cohort and 3.85 cm (IQR: 2.0–5.0) in the validation cohort (*p* = 0.440). The distributions of pT stage (*p* = 0.357), pN stage (*p* = 0.385), and pTNM stage (*p* = 0.396) were also similar between the groups. Regarding histological differentiation, the proportions of well-, moderately, and poorly differentiated tumors were comparable between cohorts (*p* = 0.812). Serum CEA levels were within normal range (≤5 ng/mL) in 80% of the training cohort and 83.9% of the validation cohort (*p* = 0.365). Similarly, the CA19-9 levels were normal (≤37 U/mL) in 82.5% and 78.0% of the patients in the training and validation cohorts, respectively (*p* = 0.287). In the present study, a total of 73 DFS events were documented in the training cohort (*n* = 275) and 36 DFS events in the independent validation cohort (*n* = 118) during the follow-up period. These findings suggest that the training and validation cohorts were well-balanced and suitable for subsequent modeling and validation.

### 3.2. Pathomics Features and Prediction Model

**Figure 2:**
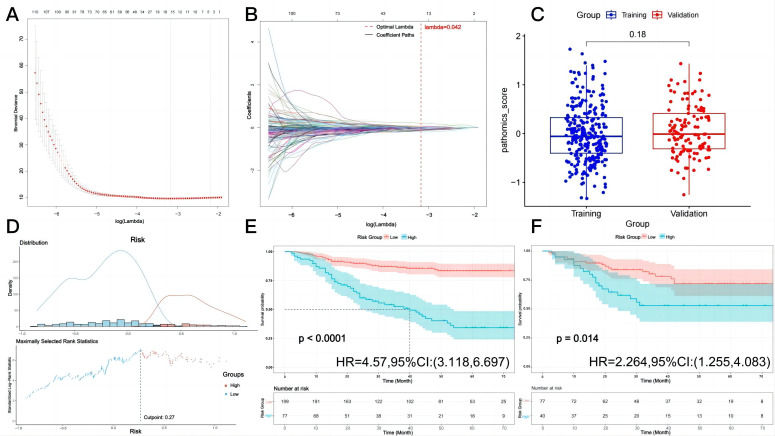
Selection of pathomics features and prognostic performance of risk model. (**A**) Ten-fold cross-validation for tuning parameter (λ) in LASSO-Cox regression model; optimal value (λ = 0.042) is indicated. (**B**) LASSO coefficient profiles of pathomics features; 16 features with non-zero coefficients were retained at optimal λ. (**C**) Pathomics scores in training and validation cohorts. (**D**) Distribution of risk scores and determination of optimal cut-off value (0.27) using maximally selected rank statistics to stratify patients into high- and low-risk groups. (**E**) Kaplan–Meier curves for disease-free survival (DFS) in training cohort, (**F**) Kaplan–Meier curves for DFS in validation cohort.

To construct a prognostic risk model, LASSO-Cox regression analysis was performed on candidate genes in the training cohort. Ten-fold cross-validation identified the optimal penalty parameter, λ = 0.042 (Figure 2A), at which point the model achieved the minimum binomial deviance. The coefficient profile plot showed that as λ increased, most gene coefficients shrank to zero, ultimately retaining a subset of key prognostic genes (Figure 2B). In total, 16 pathomics features with non-zero coefficients were selected from an initial set of 380 pathomics features, indicating their significant contribution to the model (Table 2). The pathomics scores for each patient were calculated based on the multivariate Cox regression coefficients. The average pathomics scores in the training and validation cohorts were comparable (Figure 2C). The optimal cut-off value of 0.27 was determined using maximally selected rank statistics implemented via the survminer package in R software, and this cut-off value was directly applied to the validation cohort (Figure 2D). This threshold stratified patients into high- and low-risk groups. The Kaplan–Meier survival curves showed that in the training cohort, high-risk patients exhibited significantly worse DFS compared with low-risk patients (HR = 4.57, 95% CI: 3.118–6.697, *p* < 0.0001, Figure 2E). The same trend was observed in the validation cohort, where the high-risk group had markedly poorer DFS (HR = 2.264, 95% CI: 1.255–4.083, *p* < 0.0001, Figure 2F). These findings confirmed that the constructed risk model provided strong prognostic discrimination in both the training and validation sets.

### 3.3. Clinic Factors Related to DFS

Univariable Cox regression analysis identified several clinicopathological variables significantly associated with survival (Table 3). Tumor size, pT stage, advanced N stage, pTNM stage, differentiation degree, ECOG score, and CA19-9 levels were significantly correlated with prognosis. Multivariable Cox regression analysis demonstrated that pT stage (HR = 1.42, 95% CI: 1.04–1.91, *p* = 0.027), differentiation degree (HR = 2.11, 95% CI: 1.35–3.29, *p* = 0.001), and ECOG performance status (HR = 0.586, 95% CI: 0.358–0.959, *p* = 0.034) remained independent prognostic factors. These results indicate that tumor stage, histological differentiation, and performance status are critical determinants of survival outcomes.

### 3.4. Comparison of Predictive Model Performance

**Figure 3:**
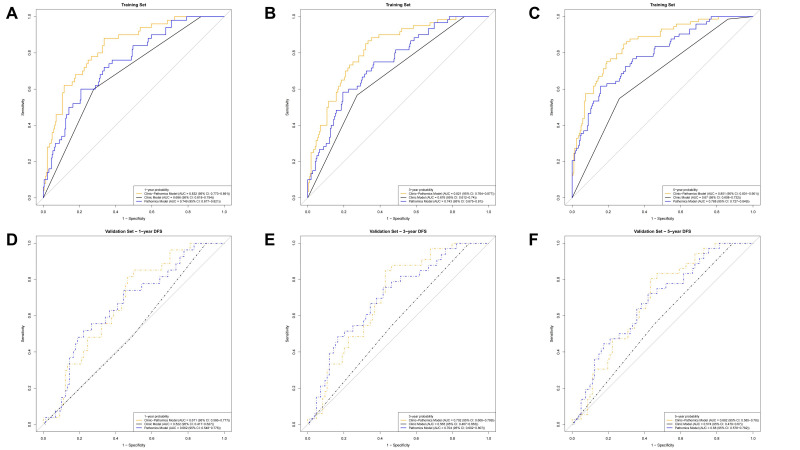
Comparison of predictive performance among three different models for 1-, 3-, and 5-year DFS. The AUCs for the three different models in (**A**–**C**) training cohort and (**D**–**F**) validation cohort.

To enhance prognostic accuracy, a clinic–pathomics model was constructed by integrating clinicopathological parameters with quantitative pathomics features. As anticipated, the clinic–pathomics model consistently outperformed both the clinic or pathomics models in discriminating survival outcomes across all time points in both the training and validation cohorts. In the training set, the clinic–pathomics model achieved AUCs of 0.832 (95% CI: 0.779–0.897), 0.821 (95% CI: 0.754–0.873), and 0.851 (95% CI: 0.792–0.895) for predicting 1-, 3-, and 5-year survival probabilities, respectively, with a C-index of 0.792 (Figure 3A–C). By comparison, the clinic-only model yielded lower AUCs of 0.686 (95% CI: 0.618–0.754), 0.676 (95% CI: 0.612–0.740), and 0.670 (95% CI: 0.608–0.732), with a C-index of 0.659, while the pathomics model achieved AUCs of 0.749 (95% CI: 0.677–0.821), 0.743 (95% CI: 0.675–0.810), and 0.788 (95% CI: 0.727–0.849), with a C-index of 0.728 at the corresponding time points. In the validation set, the clinic–pathomics model maintained superior performance, with AUCs of 0.671 (95% CI: 0.566–0.777), 0.702 (95% CI: 0.606–0.799), and 0.682 (95% CI: 0.585–0.780) for 1-, 3-, and 5-year predictions, respectively, with a C-index of 0.698 (Figure 3D–F). The clinic model demonstrated notably lower predictive accuracy, with AUCs of 0.522 (95% CI: 0.417–0.627), 0.565 (95% CI: 0.467–0.663), and 0.574 (95% CI: 0.479–0.670), with a C-index of 0.543, whereas the pathomics model achieved AUCs of 0.662 (95% CI: 0.548–0.776), 0.704 (95% CI: 0.602–0.807), and 0.680 (95% CI: 0.578–0.782), with a C-index of 0.639. Collectively, these findings confirm that integrating pathomics features with clinic variables substantially improves prognostic accuracy over either model alone, with the most pronounced gains observed in the training cohort and preserved benefits in the independent validation cohort.

### 3.5. Nomogram for Personalized Prediction of DFS

**Figure 4:**
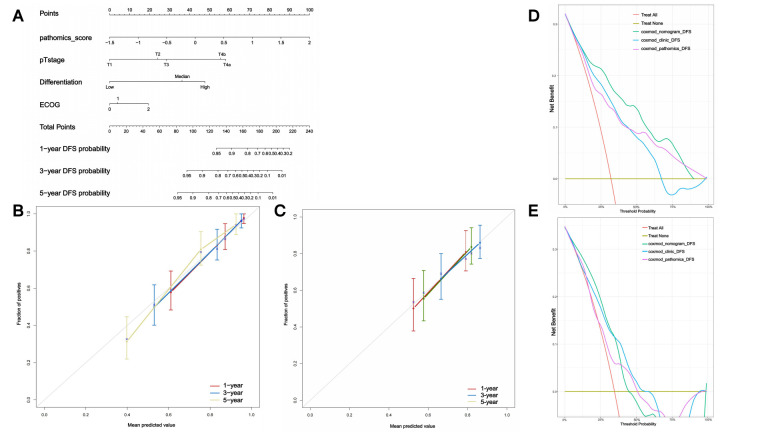
Nomogram of the clinic–pathomics model for individualized DFS prediction in GC. (**A**) Nomogram incorporating pathomics score, differentiation degree and pT stage for predicting 1-, 3-, and 5-year DFS. (**B**,**C**) Calibration curves for the nomogram in the training (**B**) and validation (**C**) cohorts, showing good agreement between predicted and observed DFS probabilities. (**D**,**E**) Decision curve analysis (DCA) for the clinic–pathomics model compared with clinic-only and pathomics-only models in the training (**D**) and validation (**E**) cohorts, demonstrating higher net benefit across a wide range of threshold probabilities.

A prognostic nomogram was constructed based on the independent predictors identified in the multivariable Cox regression analysis, including pathomics score, differentiation degree, pT stage, and ECOG stage (Figure 4A). The nomogram provides an individualized prediction of 1-, 3-, and 5-year DFS probabilities by summing the points assigned to each variable. The calibration plots demonstrate good agreement between the predicted and observed DFS probabilities in both the training set (Figure 4B) and the validation set (Figure 4C), indicating the model’s high calibration accuracy. Decision curve analysis (DCA) further showed that the clinic–pathomics nomogram yielded a higher net benefit across a wide range of threshold probabilities compared with the clinic and pathomics models in both the training (Figure 4D) and validation cohorts (Figure 4E). These findings confirm that the nomogram offers accurate and clinically useful individualized DFS prediction, with clear potential for guiding patient-specific management strategies.

## 4. Discussion

In this study, we developed and validated a prognostic risk model integrating pathomics features with clinic variables for predicting DFS in patients with GC. Using LASSO-Cox regression, we identified 16 pathomics features with non-zero coefficients that were significantly associated with DFS. The risk model effectively stratified patients into high- and low-risk groups, with the high-risk group exhibiting significantly poorer overall survival in both the training and validation cohorts. Furthermore, multivariable Cox regression analysis revealed that pT stage, histological differentiation degree, and ECOG performance status were independent prognostic factors, underscoring their clinical importance. By integrating pathomics features with clinical variables, a combined model was developed. Time-dependent ROC analysis was performed to assess the performance of the combined model over time, and the results demonstrated that the clinic-pathomics model consistently outperformed both the clinical-only and pathomics-only models in terms of predictive accuracy. The clinic–pathomics model offers a promising nomogram for personalized treatment strategies, enabling clinicians to make more informed decisions based on a broader range of data.

Although radical surgical resection remains the current method for curing GC, the risk of postoperative recurrence still exists. Postoperative adjuvant therapy is therefore essential for patients at advanced stages, such as stage III and IV, particularly those with lymph node metastasis or a risk of distant metastasis [20,21]. Park et al. [22] found that in patients with stage II/III, node-positive GC who underwent curative D2 resection, adjuvant therapy with SOX or SOXRT significantly prolonged DFS. In a separate study, Ronan J. Kelly et al. [23] explored the combination of PD-1 and LAG-3 blockade in GC as part of neoadjuvant immune checkpoint inhibition, proposing that this combination may provide a new therapeutic opportunity for future treatments. On the other hand, Lordick et al. [24] reported that the combination of nivolumab and ipilimumab did not lead to improved DFS when compared with chemotherapy in patients with ypN^+^ and/or R1 GC adenocarcinoma after receiving neoadjuvant chemotherapy and surgery. Vos et al. [25] found that, in patients undergoing surgical resection for locally advanced adenocarcinoma of the GOJ, OS and DFS did not differ significantly between patients who had neoadjuvant chemoradiation compared with chemotherapy. Thus, accurately distinguishing potential recurrence risks is crucial, and an efficient and precise DFS prediction model would play a key role in guiding patient treatment. Such a model would not only help tailor treatment strategies but also alleviate patient concerns and minimize unnecessary medical expenditures.

Various postoperative surveillance strategies have been proposed. Ho-Kyoung Lee et al. [26] classified patients with GC into two distinct gastro-type groups based on their MAM profiles, which were linked to varying risks of metachronous recurrence. Yuan et al. [27] demonstrated that residual ctDNA after adjuvant chemotherapy effectively predicted a higher risk of recurrence in stage II/III GC, and combining tissue-based with circulating tumor features offered improved risk prediction. Zhang et al. [28] identified a qualitative transcriptional signature that helps assess recurrence risk in patients with GC following surgical resection for stages II-III. These strategies provide potentially reliable tools for postoperative recurrence prediction, while also making the prediction of postoperative recurrence more complex and diverse.

Pathomics, which uses computational methods to analyze pathological images, holds great potential for predicting postoperative recurrence in those with cancer [18,29,30,31]. By extracting quantitative morphological features from tumor tissue slides, pathomics offers a detailed and objective way to assess tumor characteristics linked to recurrence risk, such as cellular morphology, tumor architecture, immune cell infiltration, and vascular patterns. These features, combined with clinical data like tumor stage and lymph node involvement, provide a comprehensive risk assessment, enhancing clinicians’ ability to predict recurrence and guide treatment strategies [32]. Moreover, advances in machine learning and artificial intelligence have made it possible to automate the extraction of pathomics features, allowing for large-scale, high-throughput analyses [10,33,34].

Notably, the 16 pathomics features identified in our study show close correlations with distinct histopathological patterns and the core biological mechanisms driving GC progression and recurrence and can be classified into five functional clusters with specific pathological implications: (1) Correlation: this feature quantifies the colocalization of eosin and hematoxylin signals, and its negative coefficient indicates a loss of the normal spatial correlation between nuclei and cytoplasm—a morphological hallmark of malignant transformation in gastric epithelial cells. (2) Granularity: this feature quantifies the spatial heterogeneity of tissue texture across different scales, and aberrant values mirror the disorganization of gastric glandular architecture, elevated nuclear pleomorphism, and uneven distribution of tumor cell nests—classic histopathological hallmarks of aggressive GC phenotypes. High granularity heterogeneity in hematoxylin channels (which target cell nuclei) indicates aberrant clonal expansion of tumor cells and loss of intercellular adhesion, key processes underlying GC invasion and metastasis. (3) Image quality: this feature characterizes the clarity, signal uniformity and maximal intensity distribution of H&E-stained images, which are direct reflections of the morphological integrity of GC tissue sections. Abnormal values of this feature are associated with blurred nuclear staining, uneven distribution of eosinophilic stroma, and poor structural definition of tumor-stroma boundaries—all of which are linked to aggressive tumor biology and reflect the disordered tissue microenvironment in GC. (4) Intensity: hematoxylin and eosin intensity features quantify the absolute staining intensity and the proportion of maximal intensity pixels in the corresponding channels. Reduced eosin intensity correlates with diminished cytoplasmic volume of tumor cells, an elevated nuclear–cytoplasmic ratio, and sparse stromal components in GC tissues; altered hematoxylin intensity reflects abnormal chromatin condensation in tumor cell nuclei, a key morphological feature of malignant proliferation. (5) Texture: as a dedicated spatial texture feature, it quantifies the non-uniformity and spatial distribution pattern of chromatin staining in hematoxylin channels. Aberrant values of this feature indicate disorganized chromatin arrangement, irregular nuclear contour, and increased nuclear heterogeneity—hallmark morphological changes of GC cells with malignant potential. Such chromatin texture abnormalities are closely associated with genomic instability (e.g., gene mutation, chromosomal rearrangement) and dysregulated DNA repair mechanisms in GC, which drive uncontrolled tumor cell proliferation and metastatic capacity.

Recent studies highlight the value of integrating pathomics into prognostic models for GC recurrence [18,30,35,36]. Wang et al. [35] showed that a radiopathomics signature, which combines both radiomics and pathomics, effectively predicts prognosis and chemotherapy benefit in patients with GC. Chen et al. [18] suggested that a radiopathomics nomogram accurately predicted the pathological response to preoperative chemotherapy, providing a valuable tool for personalized treatment in GC. Chen et al. [36] demonstrated that a nomogram incorporating both the pathomics signature and TNM staging systems significantly improves prognostic accuracy compared to the TNM staging system alone. In this study, 16 pathomics features were identified to establish a prediction model, which was then integrated with clinical factors. The ROC analysis indicated that the clinic–pathomics model had AUCs of 0.832 (95% CI: 0.779–0.897), 0.821 (95% CI: 0.754–0.873), and 0.851 (95% CI: 0.792–0.895) for 1-, 3-, and 5-year survival in the training cohort, outperforming both the clinic and pathomics models. In the independent validation cohort, the clinic–pathomics model maintained superior performance, showing strong predictive accuracy in both the training and validation cohorts. The clinic–pathomics nomogram also had higher outcome prediction accuracy, providing more precise risk stratification for personalized treatment strategies. These findings highlight the value of integrating pathomics with clinical data, which enhances predictive accuracy, supports personalized treatment, and improves overall decision-making and outcomes in GC treatment.

Several limitations should be acknowledged. First, this study was conducted retrospectively, and selection bias cannot be completely excluded. Most importantly, although our clinic–pathomics model was validated in an independent internal cohort, the absence of external validation across multi-center, geographically diverse and prospective populations constitutes a key limitation, which restricts its generalizability across institutions, ethnicities, and patients with diverse clinical features or treatment strategies—an essential prerequisite for the clinical translation and broad utility of gastric cancer prognostic models. Thus, further external validation in multi-center, prospective cohorts is warranted to confirm the model’s generalizability. Second, therapy heterogeneity introduced an unavoidable bias to this study, which, together with the inherent limitations of the single-center retrospective design, may affect the interpretability of our findings. Thus, large-sample, multi-center prospective studies are warranted in the future to validate the reliability and generalizability of our results. Third, the biological interpretation of the pathomics features remains indirect; future studies should aim to link these image-derived features to molecular and genetic profiles to better understand their mechanistic significance.

## 5. Conclusions

In conclusion, this study successfully developed and validated a clinic–pathomics model for predicting DFS in patients with GC. By integrating 16 pathomics features with clinical factors, the model demonstrated superior predictive accuracy compared to clinic-only and pathomics-only models, as shown by the ROC analysis in both the training and validation cohorts. The clinic–pathomics model not only provides a more precise risk stratification but also offers a nomogram for personalized treatment strategies, enabling clinicians to make informed decisions tailored to individual patient profiles. These findings underline the importance of integrating pathomics with clinical data to enhance prognostic accuracy and optimize treatment strategies.

## Figures and Tables

**Table 1:** Baseline characteristics of patients with GC in training and validation cohorts.

Characteristic	Training Cohort (*n* = 275)	Validation Cohort (*n* = 118)	*p*-Value
Age	63.89 ± 10.214	64.19 ± 10.037	0.794
Age, *n* (%)			0.864
≤60 years	91 (33.1)	38 (32.2)	
>60 years	184 (66.9)	80 (67.8)	
Sex, *n* (%)			0.449
Male	207 (75.3)	93 (78.8)	
Female	68 (24.7)	25 (21.2)	
Tumor size	4.0 (2.5,6.0)	3.85 (2.0,5.0)	0.440
Tumor size, (cm)			0.634
≤4 cm	156 (56.7)	70 (59.3)	
>4 cm	119 (43.3)	48 (40.7)	
T stage, *n* (%)			0.357
T1	80 (29.1)	43 (36.4)	
T2	31 (11.3)	13 (11)	
T3	60 (21.8)	19 (16.1)	
T4a	76 (27.6)	30 (25.4)	
T4b	28 (10.6)	13 (11)	
N stage, *n* (%)			0.385
N0	61 (22.2)	26 (22)	
N1	81 (29.5)	44 (37.3)	
N2	72 (26.2)	23 (19.5)	
N3a	45 (16.4)	22 (18.6)	
N3b	16 (5.8)	3 (2.5)	
pTNM Stage, *n* (%)			0.396
I	78 (28.4)	48 (40.7)	
II	76 (27.6)	15 (12.7)	
III	121 (44)	55 (46.6)	
Differentiation degree, *n* (%)			0.812
Low	26 (9.5)	12 (10.2)	
Median	143 (52.2)	62 (52.5)	
High	105 (38.4)	44 (37.3)	
ECOG, *n* (%)			0.348
0	145 (52.7)	69 (58.5)	
1	123 (44.7)	45 (38.1)	
2	7 (2.5)	4 (3.4)	
CEA (ng/mL), *n* (%)			0.365
≤5 ng/mL	220 (80)	99 (83.9)	
>5 ng/mL	55 (20)	19 (16.4)	
CA19-9 (U/mL), *n* (%)			0.287
≤37 U/mL	227 (82.5)	93 (78)	
>37 U/mL	48 (17.5)	26 (22)	

**Table 2:** The selected pathomics features.

Pathomics Features	Coefficient
Correlation_RWC_Eosin_Hematoxylin	−0.01605085
ExecutionTime_10MeasureGranularity	0.2157163
Granularity_10_Hematoxylin	−0.08096611
Granularity_15_Hematoxylin	−0.1353126
Granularity_3_Hematoxylin	0.03828404
Granularity_4_Hematoxylin	0.005451144
Granularity_4_0rigGrayHE	−0.01544835
Granularity_5_0rigGrayHE	−0.09506561
Granularity_6_Eosin	0.0893418
Granularity_7_Eosin	0.04058093
ImageQuality_LocalFocusScore_Hematoxylin_20	−0.03809315
ImageQuality_MaxIntensity_Eosin	−0.1274839
ImageQuality_PercentMaximal_Hematoxylin	0.03203714
Intensity_MaxIntensity_Eosin	−0.0465597
Intensity_PercentMaximal_Hematoxylin	1.58036 × 10^−6^
Texture_InfoMeas2_Hematoxylin_3_00_256	−0.1924062

**Table 3:** Univariate and multivariate Cox regression analyses in training cohort.

	Univariable			Multivariable		
	HR	95% CI	*p* Value	HR	95% CI	*p* Value
Age (years) (≤60 vs. >60)	0.987	0.96–1.01	0.23			
Sex (male vs. female)	1.163	0.683–1.98	0.579			
Tumor size (cm) (≤4 vs. >4)	0.2895	0.171–0.489	<0.001	1.06	0.95–1.18	0.30
pT stage			<0.001	1.42	1.04–1.91	0.027
T1	Reference		-	Reference		-
T2	3.5414	1.123–11.163	0.031	0.108	0.020–0.583	0.010
T3	3.1094	1.062–9.101	0.038	0.561	0.198–1.588	0.276
T4a	10.354	4.062–26.395	<0.001	0.367	0.158–0.854	0.020
T4b	12.338	4.477–33.998	<0.001	1.015	0.545–1.888	0.963
pN stage			<0.001	1.22	0.89–1.67	0.205
N0	Reference		-			
N1	1.66	0.521–5.293	0.392			
N2	3.97	1.350–11.674	0.012			
N3a	7.84	2.739–22.422	<0.001			
N3b	11.87	3.862–36.487	<0.001			
pTNM stage			<0.001	1.47	0.76–2.87	0.253
stage I	Reference		-			
stage II	1.944	0.74–5.109	0.177			
stage III	7.207	3.283–15.821	<0.001			
Differentiation degree			<0.001	2.11	1.35–3.29	0.001
Low	Reference		-	Reference		-
Median	7.175	0.9802–52.52	0.0523	0.100	0.013–0.745	0.025
High	18.029	2.4762–131.27	0.0043	0.585	0.350–0.977	0.041
ECOG			0.0029	0.586	0.358–0.959	0.034
0	Reference		-	Reference		-
1	2.2915	1.335–3.933	0.0026	2.080	0.828–5.225	0.119
2	0.3579	1.329–5.874	0.0067	0.785	0.375–1.644	0.520
Tumor location			0.35			
1	Reference		-			
2	0.6909	0.3415–1.398	0.304			
3	0.7016	0.3928–1.253	0.231			
4	1.8750	0.4303–8.170	0.403			
CEA (≤5 vs. >5)	0.9037	0.531–1.539	0.709			
CA19-9 (≤37 vs. >37)	0.5321	0.320–0.884	0.0148	1.525	0.902–2.577	0.115

## Data Availability

The data are available from the corresponding author on reasonable request.
